# High Mortality in HIV-Associated Cryptococcal Meningitis Patients Treated With Amphotericin B–Based Therapy Under Routine Care Conditions in Africa

**DOI:** 10.1093/ofid/ofy267

**Published:** 2018-10-23

**Authors:** Raju K K Patel, Tshepo Leeme, Caitlin Azzo, Nametso Tlhako, Katlego Tsholo, Ephraim O Tawanana, Mooketsi Molefi, Mosepele Mosepele, David S Lawrence, Margaret Mokomane, Mark W Tenforde, Joseph N Jarvis

**Affiliations:** 1 Botswana–University of Pennsylvania Partnership, Gaborone, Botswana; 2 Princess Marina Hospital Laboratory, Gaborone, Botswana; 3 University of Botswana, Gaborone, Botswana; 4 Botswana Harvard AIDS Institute Partnership, Gaborone, Botswana; 5 Department of Clinical Research, Faculty of Infectious and Tropical Diseases, London School of Hygiene and Tropical Medicine, London, UK; 6 Botswana National Health Laboratory, Gaborone, Botswana; 7 Division of Allergy and Infectious Diseases, Department of Medicine, University of Washington School of Medicine, Seattle, Washington; 8 Department of Epidemiology, University of Washington School of Public Health, Seattle, Washington; 9 Division of Infectious Diseases, Perelman School of Medicine, University of Pennsylvania, Philadelphia, Pennsylvania

**Keywords:** amphotericin B, cryptococcal meningitis, HIV, resource-limited settings, sub-Saharan Africa

## Abstract

**Background:**

Cryptococcal meningitis (CM) causes 10%–20% of HIV-related deaths in Africa. Due to limited access to liposomal amphotericin and flucytosine, most African treatment guidelines recommend amphotericin B deoxycholate (AmB-d) plus high-dose fluconazole; outcomes with this treatment regimen in routine care settings have not been well described.

**Methods:**

Electronic national death registry data and computerized medical records were used to retrospectively collect demographic, laboratory, and 1-year outcome data from all patients with CM between 2012 and 2014 at Botswana’s main referral hospital, when recommended treatment for CM was AmB-d 1 mg/kg/d plus fluconazole 800 mg/d for 14 days. Cumulative survival was estimated at 2 weeks, 10 weeks, and 1 year.

**Results:**

There were 283 episodes of CM among 236 individuals; 69% (163/236) were male, and the median age was 36 years. All patients were HIV-infected, with a median CD4 count of 39 cells/mm^3^. Two hundred fifteen person-years of follow-up data were captured for the 236 CM patients. Complete outcome data were available for 233 patients (99%) at 2 weeks, 224 patients (95%) at 10 weeks, and 219 patients (93%) at 1 year. Cumulative mortality was 26% (95% confidence interval [CI], 20%–32%) at 2 weeks, 50% (95% CI, 43%–57%) at 10 weeks, and 65% (95% CI, 58%–71%) at 1 year.

**Conclusions:**

Mortality rates following HIV-associated CM treated with AmB-d and fluconazole in a routine health care setting in Botswana were very high. The findings highlight the inadequacies of current antifungal treatments for HIV-associated CM and underscore the difficulties of administering and monitoring intravenous amphotericin B deoxycholate therapy in resource-poor settings.

HIV-associated cryptococcal meningitis (CM) causes an estimated 181 100 deaths per year, 73% of which occur in sub-Saharan Africa (SSA) [1]. Ten-week mortality in developed country settings is as low as 10%–15% with amphotericin B deoxycholate (AmB-d)–based treatment [2–5]. In contrast, even under ideal clinical trial conditions, 10-week mortality with AmB-d-based therapies in Africa is around 30%–40% [6–8]. As AmB-d requires prolonged hospitalization with daily intravenous (IV) administration and is associated with hematological, renal, and metabolic toxicities [9], many under-resourced settings instead rely on oral fluconazole. Fluconazole monotherapy is associated with worse fungal clearance and an estimated 70% mortality within 3 months [10].

Short- and long-term outcomes with AmB-d under routine care settings in SSA are not well described. Several factors are important in CM management and may affect outcomes even with highly fungicidal AmB-d, including pretreatment hydration, electrolyte supplementation and regular laboratory monitoring for AmB-d-related toxicities [11], therapeutic lumbar punctures for management of intracranial pressure [12], and initiation and timing of antiretroviral therapy (ART) [6]. As countries in SSA increasingly adopt AmB-d, as recommended in international guidelines [13, 14], usually given with high-dose fluconazole due to lack of access to flucytosine in Africa [15], a better understanding of outcomes in routine hospital settings is needed to accurately characterize morbidity and mortality from cryptococcal disease and to improve management.

Botswana, a middle-income country in Southern Africa with a 25% adult HIV prevalence and high incidence of CM [16, 17], has been using AmB-d for over a decade [18]. National treatment guidelines recommend 14-day treatment with AmB deoxycholate 1 mg/kg/d and fluconazole 800 mg/d, followed by fluconazole maintenance [19]. Utilizing Botswana’s robust electronic national death registry and computerized medical records systems, we collected clinical, laboratory, and comprehensive 1-year outcome data of patients with microbiologically confirmed CM between 2012 and 2014 at the country’s major referral hospital to assess mortality with amphotericin-based therapy under usual care conditions and explore factors associated with poor outcomes.

## METHODS

### Participants and Procedures

Data were retrospectively collected on consecutive patients admitted to Princess Marina Hospital with laboratory-confirmed CM between January 1, 2012, and December 31, 2014. Princess Marina Hospital is a 530-bed public hospital in Gaborone that serves as 1 of 2 national referral centers for Botswana, providing free treatment to all citizens. The study was conducted in accordance with the ethical standards of the Helsinki Declaration (amended in 2008) and approved by institutional review boards at the University of Pennsylvania, University of Botswana, and Princess Marina Hospital, as well as the Botswana Ministry of Health’s Health Research and Development Committee. A waiver of informed consent was obtained as we collected routine retrospective data and as individual-level patient data were anonymized for the analysis. We included all patients who had laboratory-confirmed CM by either cerebrospinal fluid (CSF) India ink stain, cryptococcal antigen (CrAg) latex agglutination test, or fungal culture, with no exclusion criteria. Patients re-admitted with a recurrence of CM symptoms and laboratory-confirmed CM at any time point after their initial admission were classified as relapse episodes. During the study period, the recommended treatment for CM was amphotericin B deoxycholate 1 mg/kg/d IV plus fluconazole 800 mg/d orally for 14 days, followed by standard fluconazole consolidation and maintenance therapy. Electrolyte supplementation was given at the responsible physician’s dicretion. Antiretroviral therapy (ART) was freely available in the hospital and at public sector clinics, with tenofovir, emtricitabine, and efavirenz as firstline.

### Evaluation and Outcomes

Cases of CM were identified through review of prospectively collected laboratory records. Demographic information and laboratory results, including blood tests, CSF results, HIV status and associated laboratory results, and basic clinical information including ART use and admission and discharge dates, were then retrospectively obtained for all patients from the electronic medical records system. More detailed clinical history and examination findings, CSF opening pressure recordings, and CM treatment details were obtained from detailed review of paper hospital records, where available, using a standardized data collection form. Time from hospital triage to initiation of antifungal therapy, duration and cumulative dosage of antifungal therapies, number of missed drug doses, frequency of toxicity monitoring, and details of management of raised intracranial pressure were recorded for each CM episode. Mortality data for all patients were collected by linking laboratory records to the electronic national death registry at the Botswana Ministry of Labour and Home Affairs using unique individual identifiers (name, date of birth, and Omang [national identification number]) at the end of December 2015. Cumulative mortality at 2 weeks, 10 weeks, and 1 year after hospital admission with CM were recorded.

### Statistical Analysis

Baseline demographic, clinical, and laboratory variables were summarized using descriptive statistics (counts and percentages, medians and interquartile ranges [IQRs]), and proportions were compared using χ^2^ testing. Cumulative survival was estimated at 2 weeks, 10 weeks, and 1 year after initial admission for all patients (ie, from the first admission if a patient had multiple episodes of CM) and displayed graphically on a Kaplan-Meier curve. Sensitivity analyses were performed assuming all missing patients either survived or died at the point of censoring. Cox proportional hazards models were constructed to explore factors associated with mortality. Unadjusted and adjusted hazard ratios were estimated for predictors of interest, and *P* values were derived using the likelihood ratio test. Statistical significance was defined as a *P* value ≤.05. Analyses were performed using Stata, version 13 (College Station, TX).

## RESULTS

During the study period, there were 283 episodes of CM among 236 individuals (199 patients had a single episode, and 37 patients had 2 or more episodes a median [IQR] of 133 [23–614] days apart) (Table 1). The median age (IQR) was 36 (32–42) years, and 69% (163/236) of patients were male. All patients were HIV-infected, with a median baseline CD4 count (IQR) of 39 (17–83) cells/mm^3^. Seventy-five percent (177/236) of patients had documentation of HIV infection before their initial CM admission, diagnosed a median (IQR) of 14 (2–63) months earlier. Of the known HIV-infected individuals with treatment data available, 57% (81/141) were taking ART at admission, started a median (IQR) of 31 (6–156) weeks before. Baseline laboratory data were available for all patients (Table 1). Eighty-nine percent (210/236) were India ink–positive at first presentation. The median CSF white cell count (IQR) was 10 (0–62) cells/µL, with a median lymphocyte predominance (IQR) of 98% (90%–99%). Paper clinical records were retrieved for 64% (180/283) of CM episodes. Headache was the most frequently recorded presenting symptom, documented in 86% (n = 154) of all CM episodes and present for a median (IQR) of 7 (5–14) days before admission. Forty-two percent (n = 74) of individuals had abnormal mental status at presentation (defined as a Glasgow Coma Score [GCS] <15). CSF opening pressure (OP) was documented in 62% (n = 112) of episodes, with a median OP (IQR) of 39 (30–55) cm H_2_O. Other common documented presenting symptoms were vomiting (55%, n = 96) and visual disturbances (37%, n = 65). Comorbidities were common, with a history of treated tuberculosis (TB) in 27% of cases (n = 48) and concurrent TB treatment in 9% (n = 16).

**Table 1.** Baseline Characteristics

Cryptococcal Meningitis: Patients and Episodes		
Total No. of patients	236	
Total No. of episodes	283	
1	236	(83%)
2	37	(13%)
3	8	(3%)
4	2	(1%)
Variable	No. With Data (of 236 Patients)	Value, Median (IQR) Unless Otherwise Stated
Baseline characteristics of patients (restricted to 1st episode of cryptococcal meningitis)		
Age, y	234	36 (32–42)
Male sex, % (No.)	236	69 (163)
Headache, % (No.)^a^	155	86 (134)
Vomiting, % (No.)^a^	151	54 (81)
Visual disturbance, % (No.)^a^	155	35 (54)
Symptom duration, d^a^	119	7 (5–14)
Glasgow Coma Scale score <15, % (No.)^a^	155	40 (62)
Focal neurology, % (No.)^a^	154	14 (21)
CSF opening pressure, cm H_2_O^a^	94	38 (28–48)
India ink, % positive (No.)	235	89 (210)
CSF protein, g/dL	194	0.74 (0.47–1.38)
CSF glucose, mmol/L	195	2.3 (1.5–2.9)
CSF white cell count, cells/µL	229	10 (0–62)
CSF lymphocyte, %	105	98 (90–99)
HIV and TB status		
Prior diagnosis of HIV, % (No.)	236	75 (177)
Time from HIV diagnosis to CM, mo	217	14 (2–63)
On ART at CM diagnosis, % (No.)	181	45 (81)
Time from ART initiation to CM, d	181	217 (40–1095)
Baseline CD4 cell count, cells/µL	192	39 (17–82)
Previously treated for TB, % (No.)^a^	152	26 (39)
Current on TB treatment, % (No.)^a^	151	9 (13)

Abbreviations: ART, antiretroviral therapy; CM, cyptococcal meningitis; CSF, cerebrospinal fluid; IQR, interquartile range; TB, tuberculosis.

^a^These data were derived from paper records. Paper records were retrieved for 64% (180/283) of CM episodes, or 66% (156/236) of patients. Note that the results in the table are restricted to the *first episode only* in individuals who had more than 1 episode of CM. The overall results of these paper record variables for each episode of CM (ie, including both first and relapse episodes) are given in the text.

### Treatment and Monitoring

All patients were treated with AmB-d (at a dose of approximately 0.7–1 mg/kg/d) plus fluconazole 800 mg/d during the study period. Patient weights were not reliably documented in the medical notes, and the majority of patients (81%) received a dose of 50 mg/d, equating to a single vial of AmB-d. There was a median (IQR) of 2 (1–3) days from presentation to administration of the first AmB-d dose (Table 2). Over half of patients missed at least 1 dose of AmB-d; 20% missed a single dose, 12% missed 2 doses, and 21% missed 3 or more doses. Baseline full blood count (FBC) and renal function were available for 91% (n = 257) and 87% (n = 245), respectively. Patients had a median (IQR) of 1 (0–1) monitoring (ie, post-baseline) FBC and 2 (1–3) monitoring renal function and electrolyte tests during admission. Even with this infrequent monitoring, Division of AIDS (DAIDS) [20] Grade 3 anemia (hemoglobin [Hb] < 7.5 g/dL) was observed in 46 cases (29% of those tested), and Grade 4 anemia (Hb < 6.5g/dL) was observed in 27 cases (17% of those tested). Grade 3 creatinine rises (216–400 µmol/L/2.47–4.42 mg/dL) were observed in 22 cases (10% of those tested), and Grade 4 creatinine rises (>400 µmol/L/>4.42 mg/dL) were observed in 3 cases (1%). Therapeutic lumbar punctures were documented in 46% (129/283) of cases.

**Table 2.** Treatment and Outcomes

Variable		No. With Data (of 283 Episodes)	Value, Median (IQR) or % (No.)
Duration of admission, d		270	17 (11–22)
Time from admission to 1st amphotericin dose, d		159^a^	
	0		11 (18)
	1		36 (57)
	2+		53 (84)
No. of missed amphotericin doses^a,b^		159^a^	
	0		47 (74)
	1–2		32 (51)
	3+		21 (34)
Baseline CSF opening pressure recorded		175	64 (112)
No. of therapeutic lumbar punctures^c^			
	0	283	54 (154)
	1		16 (46)
	2		11 (32)
	3		8 (23)
	4+		10 (28)
No. of monitoring full blood counts^c^		283	1 (0–1)
Median drop in hemoglobin, g/dL^c^		146	2.0 (1.0–3.4)
Median nadir hemoglobin, g/dL^c^		161	8.9 (7.3–11.1)
DAIDS Grade 3 anemia, <7.5 g/dL^c^		161	29 (46)
DAIDS Grade 4 anemia, <6.5 g/dL^c^		161	17 (27)
No. of monitoring electrolyte tests^c^		283	2 (1–2)
Median nadir serum potassium, mmol/L^c^		222	3.2 (2.8–3.9)
DAIDS Grade 3 hypokalemia^c^		222	9 (21)
DAIDS Grade 4 hypokalemia^c^		222	0 (0)
No. of monitoring creatinine tests^c^		283	2 (1–3)
Median peak creatinine, µmol/L^c^		223	105 (73–147)
Median % rise in creatinine^c^		194	53 (9–117)
DAIDS Grade 3 creatinine rise^c^		223	10 (22)
DAIDS Grade 4 creatinine rise^c^		223	1 (3)
Outcomes^d^			
Mortality at 2 wk, % (No.)		233	26 (60)
Mortality at 10 wk, % (No.)		224	50 (112)
Mortality at 1 y, % (No.)		219	65 (142)

Abbreviations: CSF, cerebrospinal fluid; DAIDS, Division of AIDS; IQR, interquartile range.

^a^These data were derived from paper records. Paper records were retrieved for 64% (180/283) of CM episodes, or 66% (156/236) of patients.

^b^The number of missed doses was calculated from the first dose, by subtracting the actual number of doses given over 14 days from the recommended 14 doses, or, if the patient died before 14 doses, by subtracting the actual number of doses given from the number of days a patient was alive and should have received a dose.

^c^These data were derived from electronic laboratory records. Note that the number of therapeutic lumbar punctures may be underestimated as CSF may not always have been sent to the laboratory for analysis, although it is routine hospital practice to do so.

^d^Reported from date of first episode.

Missed AmB-d doses were more frequent in patients developing DAIDS Grade 4 anemia during treatment (47% [8/17] of whom missed 3 or more doses vs 15% [13/84] of those without; *P* < .01) and Grade 3 hypokalemia (44% [7/17] of whom missed 3 or more doses vs 17% [20/119] of those without; *P* = .01), but not in patients developing DAIDS Grade 3 or 4 renal impairment.

### Mortality

**Figure 1.**
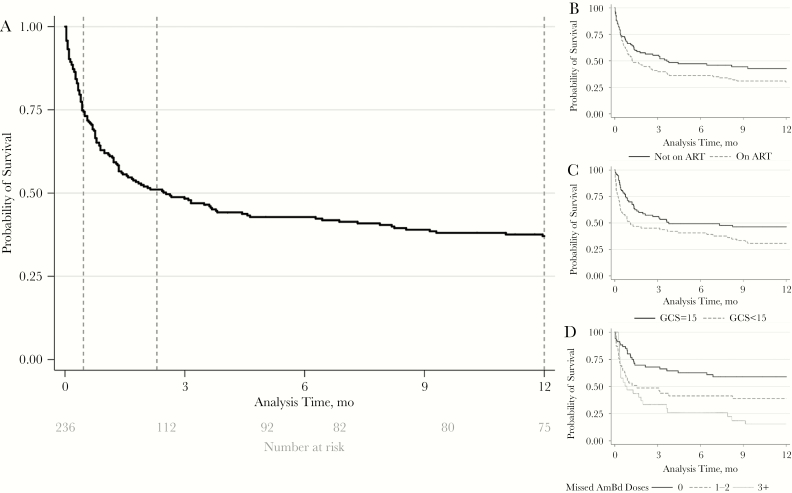
Kaplan-Meier survival curves showing the probability of survival following presentation with a first episode of HIV-associated cryptococcal meningitis in Botswana. A, Survival for all 236 patients, with vertical dotted lines indicating 2 weeks (26% mortality), 10 weeks (50% mortality), and 12 months (65% mortality). B, Survival stratified by antiretroviral therapy (ART) status at presentation. C, Survival stratified by mental status. D, According to number of missed amphotericin B deoxycholate doses.

Two hundred fifteen person-years of follow-up data were captured for the 236 CM patients. Complete outcome data were available for 233 patients (99%) at 2 weeks, 224 patients (95%) at 10 weeks, and 219 patients (93%) at 1 year after initial presentation with CM. Overall mortality was 26% (95% confidence interval [CI], 20%–32%) at 2 weeks, 50% (95% CI, 43%–57%) at 10 weeks, and 65% (95% CI, 58%–71%) at 1 year (Figure 1). In sensitivity analysis, assuming all patients missing outcome data were either alive or dead, the point estimates for mortality were 25% (60/236) and 27% (63/236), respectively, at 2 weeks; 47% (112/236) and 53% (124/236), respectively, at 10 weeks; and 60% (142/236) and 67% (159/236), respectively, at 1 year (Table 2).

### Associations With Mortality

Baseline factors significantly associated with 2-week and 10-week mortality in univariable analysis were abnormal mental status (GCS < 15), older age (>50 years), anemia (<7.5 g/dL), raised peripheral white cell count (>10 × 10^9^/L), renal impairment (creatinine >110 µmol/L/>1.24 mg/dL), hyponatremia (<125 mmol/L), and low CSF white cell count (<20 cells/µL) (Table 3A). There was no evidence for lower mortality in ART-experienced patients (Table 3A, Figure 1B) despite ART-experienced patients having significantly higher baseline CD4 counts (Supplementary Table 1). In multivariable analysis, abnormal mental status, older age, raised peripheral white cell count, hyponatremia, and low CSF white cell count remained independently associated with acute mortality (Table 3B). ART status was not associated with 2-week mortality, but ART-experienced patients had significantly higher hazards of death at 10 weeks and 1 year. Relapse episodes were associated with lower rates of India ink positivity, higher CSF white cell counts, higher CD4 counts, and lower acute mortality than initial episodes (0% vs 26% at 2 weeks and 9% vs 50% at 10 weeks; *P* < .01 for both comparisons) (Supplementary Table 2). Missing AmB-d doses during induction treatment was associated with significantly higher mortality at 2 weeks, 10 weeks, and 1 year (at 2 weeks: hazard ratio [HR], 3.20; 95% CI, 1.4–7.1; at 10 weeks: HR, 2.30; 95% CI, 1.4–3.9; at 1 year: HR, 2.33; 95% CI, 1.4–3.8) (Table 3 and Figure 1D). This association remained significant in an analysis limited to individuals who did not develop DAIDS Grade 3 anemia, hypokalemia, or renal impairment during treatment, that is, the 3 potential dose-limiting toxicities leading to AmB-d discontinuation (at 2 weeks: HR, 7.30; 95% CI, 0.9–60.0; at 10 weeks: HR, 4.55; 95% CI, 1.3–16.3; at 1 year: HR, 2.77; 95% CI, 1.1–7.3).

**Table 3.** Associations With Mortality

3A. Univariable Associations												
Variable	Category	Data, No. (%)	No. (%)	Mortality 2 wk, % (No.)	HR (95% CI)	*P* Value	Mortality 10 wk, % (No.)	HR (95% CI)	*P* Value	Mortality 1 y, % (No.)	HR (95% CI)	*P* Value
Age	<50 y	234 (99)	213 (91)	23 (49)	1	.05	47 (95)	1	<.01	61 (121)	1	<0.01
	≥50 y		21 (9)	45 (9)	2.02 (1.0–4.1)		75 (15)	2.18 (1.3–3.8)		95 (19)	2.53 (1.6–4.1)	
Sex	Male	236 (100)	163 (69)	30 (48)	1	.04	53 (82)	1	.12	64 (96)	1	0.75
	Female		73 (31)	17 (12)	0.52 (0.3–1.0)		43 (30)	0.72 (0.5–1.8)		67 (46)	0.94 (0.7–1.3)	
Mental status	GCS = 15	155 (66)	93 (60)	20 (18)	1	<.01	46 (39)	1	.04	63 (52)	1	0.02
	GCS < 15		62 (40)	39 (24)	2.35 (1.3–4.3)		53 (32)	1.64 (1.0–2.6)		66 (90)	1.64 (1.1–2.5)	
Concurrent TB	No	151 (65)	138 (91)	26 (36)	1	.19	50 (64)	1	.25	66 (83)	1	0.19
	Yes		13 (9)	46 (6)	1.7 (0.7–4.2)		54 (7)	1.54 (0.74–3.2)		69 (9)	1.55 (0.8–3.0)	
ART status	No ART	181 (77)	100 (55)	26 (26)	1	.77	46 (43)	1	.15	66 (59)	1	0.10
	On ART		81 (45)	28 (23)	1.09 (0.6–1.9)		55 (43)	1.36 (0.9–2.1)		66 (51)	1.37 (0.9–2.0)	
CD4 category	Per 50 cells/µL increase	192 (81)	192	—	0.93 (0.8–1.1)	.51	—	0.86 (0.7–1.0)	.08	—	0.87 (0.8–1.0)	0.06
Hemoglobin	≥7.5 g/dL	215 (91)	198 (92)	25 (49)	1	.13	49 (93)	1	.03	63 (119)	1	0.09
	<7.5 g/dL		17 (8)	44 (7)	1.84 (0.8–4.1)		73 (11)	1.99 (1.1–3.6)		73 (11)	1.68 (0.9–3.1)	
White blood cell count	≤10^9^/L	215 (91)	193 (90)	24 (46)	1	.01	49 (90)	1	.03	64 (115)	1	0.12
	>10^9^/L		22 (10)	45 (10)	2.44 (1.2–4.8)		64 (14)	1.87 (1.1–3.2)		68 (15)	1.52 (0.9–2.6)	
Creatinine	≤110 µmol/L	201 (85)	181 (90)	23 (41)	1	<.01	49 (84)	1	<.01	64 (108)	1	<0.01
	>110 µmol/L		20 (10)	50 (10)	2.60 (1.3–5.2)		79 (15)	2.48 (1.5–4.2)		84 (16)	2.30 (1.4–3.8)	
Sodium	≥125 mmol/L	198 (84)	166 (84)	23 (38)	1	.03	48 (76)	1	.03	63 (97)	1	0.01
	<125 mmol/L		32 (16)	44 (14)	1.99 (1.1–3.7)		66 (21)	1.69 (1.0–2.7)		77 (24)	1.76 (1.1–2.7)	
CSF OP	<30 cmH_2_O	94 (40)	27 (29)	30 (8)	1	.98	54 (14)	1	.95	72 (18)	1	0.98
	≥30 cmH_2_O		67 (71)	30 (20)	1.01 (0.4–2.3)		54 (34)	1.01 (0.5–1.9)		68 (42)	0.99 (0.6–1.8)	
CSF WCC	≥20 cells/µL	236 (100)	95 (40)	19 (18)	1	.05	39 (34)	1	<.01	62 (52)	1	0.08
	<20 cells/µL		141 (60)	30 (42)	1.73 (1.0–3.0)		57 (78)	1.80 (1.2–2.7)		67 (90)	1.36 (1.0–1.9)	
Time to first AmB-d dose	0 d	157 (67)	17 (11)	17 (3)	1	.63	29 (5)	1	.29	53 (8)	1	0.34
	1 d		56 (36)	21 (12)	1.18 (0.4–4.2)		44 (24)	1.83 (0.6–5.3)		55 (30)	1.24 (0.5–2.8)	
	2+		84 (53)	22 (18)	1.32 (0.4–4.5)		44 (34)	1.93 (0.7–5.5)		65 (49)	1.42 (0.6–3.2)	
Missed AmB-d doses^a^	0	158 (67)	74 (47)	11 (8)	1	<.01	31 (20)	1	<.01	49 (31)	1	<0.01
	1–2		50 (32)	28 (14)	2.92 (1.2–7.0)		48 (24)	2.06 (1.1–3.8)		61 (30)	1.97 (1.1–3.4)	
												
	3+		34 (21)	32 (11)	3.67 (1.5–9.1)		56 (19)	2.71 (1.4–5.1)		79 (26)	2.99 (1.7–5.2)	
3B. Multivariable Associations												
Variable	Category		2-wk Mortality-Adjusted HR			*P* Value	10-wk Mortality-Adjusted HR		*P* Value	1-y Mortality-Adjusted HR		*P* Value
Age	<50 y		1 (base)			.05	1 (base)		.03	1 (base)		0.04
	≥50 y		2.82 (1.0–8.0)				3.34 (1.1–9.7)			2.43 (1.0–5.6)		
Mental status	GCS = 15		1 (base)			<.01	1 (base)		.02	1 (base)		0.03
	GCS < 15		2.83 (1.4–5.6)				2.02 (1.1–3.7)			1.66 (1.0–2.6)		
3B. Multivariable Associations												
Variable	Category		2-wk Mortality-Adjusted HR			*P* Value	10-wk Mortality-Adjusted HR		*P* Value	1-y Mortality-Adjusted HR		*P* Value
ART status	No ART		—			—	1 (base)		<.01	1 (base)		<0.01
	On ART		—				2.16 (1.2–3.9)			1.88 (1.2–3.0)		
CD4 category	Per 50 cells/µL increase		—			—	0.83 (0.7–1.1)		.07	—		—
White blood cell count	≤10^9^/L		1 (base)			<.01	1 (base)		.04	—		—
	>10^9^/L		4.23 (1.8–9.9)				2.47 (1.1–5.8)			—		
Sodium	≥125 mmol/L		1 (base)			.05	1 (base)		.01	1 (base)		<0.01
	<125 mmol/L		2.24 (1.0–4.9)				2.79 (1.3–6.2)			2.74 (1.5–5.0)		
CSF WCC	≥20 cells/µL		1 (base)			.02	—		—	—		—
	<20 cells/µL		2.39 (1.1–5.0)				—			—		

Abbreviations: AmB-d, amphotericin B deoxycholate; ART, antiretroviral therapy; CI, confidence interval; CSF, cerebrospinal fluid; GCS, Glasgow Coma Scale score; HR, hazard ratio; OP, opening pressure; WCC, white cell count.

^a^When restricted to individuals who did not develop either Grade 3 anemia, hypokalemia, or renal impairment during amphotericin B treatment, the hazard ratios were 7.30 (95% CI, 0.9–60.0) at 2 weeks, 4.55 (95% CI, 1.3–16.3) at 10 weeks, and 2.77 (95% CI, 1.1–7.3) at 1 year.

## DISCUSSION

Mortality following HIV-associated cryptococcal meningitis in a routine health care setting in Botswana was very high, with only 35% of patients surviving to 1 year. The comprehensive national death registration system in Botswana, combined with an electronic medical records system, provided the unique opportunity to accurately determine long-term outcomes in a large observational cohort of patients treated with amphotericin B deoxycholate therapy given with high-dose fluconazole under “real-world” routine care conditions in sub-Saharan Africa. These poor patient outcomes, in 1 of the better-resourced health care systems in Africa with good access to antiretroviral therapy, highlight the inadequacies of current antifungal treatments for HIV-associated cryptococcal meningitis. The findings also underscore the difficulties faced by busy clinicians in resource-poor settings in administering and monitoring 14 days of intravenous amphotericin B deoxycholate therapy and managing the frequent drug-associated toxicities, with the majority of patients receiving incomplete treatment courses.

Mortality rates in this cohort of patients with HIV-associated cryptococcal meningitis were substantially higher than those reported in recent randomized clinical trials (RCTs) from low-resource settings [8, 21, 22]. Ten-week mortality in patients treated with amphotericin B plus fluconazole in trial settings has ranged from 33% to 41% [8, 21, 22], compared with 50% (95% CI, 43%–57%) in this routine care cohort. Although some of this observed difference may reflect the selection and survival bias inherent in clinical trials, where the sickest patients may either die before study enrollment or be too unwell to consent to study inclusion, it is likely that the more intensive nursing, medical care, and toxicity monitoring in clinical trials leads to more regular drug administration, enhanced management of raised intracranial pressure, and earlier recognition and management of amphotericin B–related toxicities—all potentially contributing to improved survival [11, 12, 23, 24]. A large proportion of deaths occurred in the early posthospitalization period, emphasizing the need for close outpatient monitoring of patients following initial cryptococcal meningitis treatment.

In this routine care cohort, toxicity monitoring was performed less frequently than recommended in World Health Organization guidelines [14], with a median of 1 postbaseline full blood count and 2 postbaseline creatinine and electrolyte tests performed per admission, similar to recent laboratory-based surveillance data from Gauteng Province, South Africa, in which once-weekly laboratory monitoring of hemoglobin, potassium, and creatinine was performed in less than 50% of patients [25]. Even with this limited monitoring, which almost certainly led to a marked underascertainment of drug-related toxicities, amphotericin B–related adverse events were frequently detected, indicating the substantial toxicity of amphotericin B deoxycholate. As expected, the main toxicities were anemia (median 2-g/dL drop in hemoglobin during treatment), renal impairment, and hypokalemia. Amphotericin B doses were frequently missed, with more than half of patients missing at least 1 dose. Although we cannot ascertain for certain from our data, it is likely that while some missed amphotericin doses were intentionally withheld due to drug-related toxicities, the majority were missed due to the logistical challenges of administering daily intravenous therapy in an under-resourced health care setting. Unlike many African settings, Princess Marina Hospital has not had regular stock-outs of amphotericin B either during the study period or subsequently.

Importantly, short-term outcomes observed with amphotericin B deoxycholate plus fluconazole 800 mg/d treatment under usual care conditions were only marginally better than the survival described in prospective cohorts and RCTs from sub-Saharan Africa with high-dose (800–1200 mg/d) fluconazole monotherapy [26–29], likely reflecting the trade-off between more effective fungal clearance with amphotericin B deoxycholate [7, 30] and greater risk of severe drug-related toxicities and additional risks with extended intravenous therapy [9, 31].

The baseline predictors of mortality in this routine care cohort—older age, abnormal mental status, raised peripheral white blood cell count, hyponatremia, and low CSF white blood cell count—have previously been described in prospective cohort studies [7]. Seventy-five percent of patients had been diagnosed with HIV before admission, and almost half (45%) were on ART at the time of admission, reflecting recent shifts in the epidemiology of cryptococcal meningitis [8, 32, 33]. In contrast to a previous smaller study from Botswana [34], there was no evidence that being on ART at the time of initial presentation with cryptococcal meningitis led to improved outcomes. Conversely, although there was no significant difference in 2-week mortality between ART-experienced and ART-naïve patients, 10-week and 1-year outcomes were significantly worse in ART-experienced individuals in adjusted analysis. This may reflect the fact that many of these individuals were already failing or defaulting ART at the time of initial presentation and had worse adherence and outcomes once re-initiated on ART.

Given the high ongoing burden of HIV-associated cryptococcal meningitis in Africa [1, 16] and the extremely poor outcomes now described with amphotericin B deoxycholate plus fluconazole treatment, there is an urgent need to improve access to more effective, safe, and easily administered cryptococcal treatments in Africa. One potential strategy is shorter-course AmB-d treatment courses (7 days) [22], which may provide equivalent fungicidal activity to standard 14-day courses while avoiding many of the associated toxicities [35, 36]. In the recently completed ACTA trial [22], 1 week of AmB-d given with flucytosine was a highly effective and well-tolerated treatment, leading to lower mortality than standard 2-week treatment courses. One-week AmB-d given with fluconazole 800 mg/d was far less effective, leading to higher mortality than standard treatment, suggesting that such a short-course strategy is only viable with a more effective oral backbone therapy than fluconazole. Compatible with these trial findings, in this routine care cohort, missing any amphotericin doses was a strong predictor of mortality, even when accounting for related dose-limiting toxicities that may have led to the doses being withheld. This supports updated guidance recommending that in the context of fluconazole as the partner drug, AmB-d treatment should be given for a full 14-day course [14], and emphasizes the urgent need to establish access to flucytosine in Africa.

Another promising strategy to reduce mortality, currently being evaluated in a phase III study (ISRCTN 72509687), is administration of single high dose of liposomal amphotericin B. Phase II data have shown single 10-mg/kg doses to be safe, with similar early fungal clearance compared with 2 weeks of standard liposomal amphotericin B dosing [37]. In parallel with efforts to develop new improved drug treatment regimens and ensure access to these treatments, work is needed to ensure that patients who are receiving the currently available amphotericin B deoxycholate treatments in low-resource settings receive adequate toxicity monitoring and routine therapeutic lumbar punctures. These factors are often related to broader health system challenges and require addressing complex issues of equipment stock-out, physician training, and health care funding.

Our study has several important limitations, primarily relating to the retrospective data collection. Botswana’s electronic death registry and electronic laboratory records enabled near complete ascertainment of 1-year vital status data and laboratory results. However, we were still dependent on retrospective medical record review to collate several key clinical variables. Paper medical records were missing in a substantial proportion of patients, limiting clinical data availability. Patient weights were rarely available, precluding any assessment of potential weight-based underdosing or overdosing of amphotericin. The median weight in the cryptococcal meningitis patients in our clinical trials in these settings (IQR) was 52 (45–61) Kg [37], with a very small minority over 70 Kg, suggesting that a single 50-mg vial should provide a suitable weight-based dose of 0.7–1 mg/kg in most cases, with a higher probability of over- rather than underdosing. Documentation of therapeutic lumbar punctures and follow-up CSF opening pressures was poor, making a full analysis of the contribution of CSF pressure management difficult. Potential drug toxicities not captured through laboratory testing, such as thrombophlebitis, vomiting, and infusion reactions, were not routinely recorded; thus we can make no inference about their frequency or how often they contributed to discontinuation of amphotericin treatment. HIV viral load data were not available for the majority of ART-experienced patients at the time of CM presentation. In addition, data regarding ART initiation post–cryptococcal treatment were not available, precluding any assessment of the impact of ART timing on long-term outcome.

In conclusion, through analysis of registry-based data and electronic medical records, this study has demonstrated the extremely high mortality resulting from HIV-associated cryptococcal meningitis in patients treated with standard amphotericin B deoxycholate and high-dose fluconazole therapy in Africa. Much of this mortality occurs in the first months after hospital discharge and would be missed in hospital-based studies. These findings highlight the urgent need for effective implementation of cryptococcal prevention strategies such as cryptococcal antigen screening, the need for new, easy-to-administer, and effective treatment regimens for cryptococcal meningitis, and the importance of advocacy efforts to ensure that drugs such as flucytosine and liposomal amphotericin B, which will form the key components of these new treatment regimens, are made accessible and affordable in Africa.

## Supplementary Data

Supplementary materials are available at *Open Forum Infectious Diseases* online. Consisting of data provided by the authors to benefit the reader, the posted materials are not copyedited and are the sole responsibility of the authors, so questions or comments should be addressed to the corresponding author.

Supplementary Tables
