# Supplementary material for: High Mortality in HIV-Associated Cryptococcal Meningitis Patients Treated With Amphotericin B–Based Therapy Under Routine Care Conditions in Africa
Source: Open Forum Infect Dis. 2018 Oct 23;5(11):ofy267. doi: 10.1093/ofid/ofy267 (PMC6251350; doi:10.1093/ofid/ofy267)
Supplement: Supplementary Tables [file ofy267_suppl_supplementary_tables.docx]

**Supplementary Table s1.**

| **Cryptococcal Meningitis (CM): Characteristics and outcomes according to ART status at presentation** | | | |
| --- | --- | --- | --- |
| **Variable** | **Not on ART**  *(n=100)* | **On ART**  *(n=81)* | ***p-value*** |
| *Baseline Characteristics of Patients (restricted to 1st episode of cryptococcal meningitis). (median [IQR] or % [N])* | | | |
| Age (years) | 38 (32-43) | 35 (31-41) | 0.11 |
| Sex (% male, N) | 67% (67) | 80% (65) | 0.05 |
| Glasgow Coma Score <15 (%, N)* | 41% (35) | 41% (26) | 0.99 |
| CSF Opening Pressure (cm H_2_O)* | 36 (30-46) | 38 (22-55) | 0.82 |
| India ink (% positive, N) | 92% (92) | 86% (69) | 0.21 |
| CSF protein (g/dL) | 0.74 (0.48-1.16) | 0.80 (0.53-1.73) | 0.34 |
| CSF glucose (mmol/L) | 2.4 (1.7-2.9) | 2.0 (1.3-3.0) | 0.22 |
| CSF white cell count (cells/µL) | 10 (0-65) | 10 (0-85) | 0.82 |
| *HIV and TB Status* |  |  |  |
| Baseline CD4 cell count (cells/µL) | 35 (17-62) | 63 (17-134) | <0.01 |
| Previously treated for TB (%, N)* | 18% (15) | 34% (22) | 0.03 |
| Current on TB treatment (%, N)* | 6% (5) | 13% (8) | 0.16 |
| *Outcomes* | | | |
| Mortality at 2 weeks (%, N) | 26% (26) | 28% (23) | 0.75 |
| Mortality at 10 weeks (%, N) | 46% (43) | 56% (43) | 0.19 |
| Mortality at 1 year (%, N) | 66% (59) | 66% (51) | 0.93 |

*Restricted to those with paper records only

**Supplementary Table s2.**

| **Cryptococcal Meningitis (CM): Characteristics and outcomes in initial versus relapse presentations** | | | |
| --- | --- | --- | --- |
| **Variable** | **First episode**  *(n=236)* | **Relapse episode**  *(n=47)* | ***p-value*** |
| *Baseline Characteristics of Patients. (median [IQR] or % [N])* | | | |
| Age (years) | 36 (32-42) | 39 (33-43) | 0.53 |
| Sex (% male, N) | 69% (163) | 57% (27) | 0.12 |
| Glasgow Coma Scale score <15 (%, N)* | 40% (62) | 52% (12) | 0.27 |
| CSF opening pressure (cm H_2_O)* | 38 (28-48) | 52 (46-55) | <0.01 |
| India ink (% positive, N) | 89% (210) | 77% (36) | <0.01 |
| CSF protein (g/dL) | 0.74 (0.47-1.38) | 1.02 (0.65-1.44) | 0.21 |
| CSF glucose (mmol/L) | 2.3 (1.5-2.9) | 2.1 (1.1-2.9) | 0.42 |
| CSF white cell count (cells/µL) | 10 (0-62) | 25 (0-80) | 0.42 |
| *HIV and TB Status* |  |  |  |
| On ART at CM diagnosis (%, N) | 45% (81) | 87% (27) | <0.01 |
| Baseline CD4 cell count (cells/µL) | 39 (17-82) | 79 (19-118) | 0.02 |
| Previously treated for TB (%, N)* | 26% (39) | 41% (9) | 0.14 |
| Current on TB treatment (%, N)* | 9% (13) | 14% (3) | 045 |
| *Outcomes* | | | |
| Mortality at 2 weeks (%, N) | 26% (60) | 0% (0) | <0.01 |
| Mortality at 10 weeks (%, N) | 50% (112) | 9% (4) | <0.01 |
| Mortality at 1 year (%, N) | 65% (142) | 43% (47) | <0.01 |

*Restricted to those with paper records only
